# Associations of Biomarkers and Body Water with Dengue Status and Length of Hospital Stay: A Single-Center Observational Study

**DOI:** 10.3390/pathogens15050501

**Published:** 2026-05-06

**Authors:** Thang Van Dao, Binh Nhu Do, Minh Duc Pham, Duc Minh Cap, Kien Trung Nguyen, Tuyen Van Duong

**Affiliations:** 1 International Ph.D. Program in Medicine, College of Medicine, Taipei Medical University, Taipei 11031, Taiwan; d142111016@tmu.edu.tw; 2 Department of Infectious Diseases, Military Hospital 103, Vietnam Military Medical University, Hanoi 12108, Vietnam; 3 Department of Military Science, Vietnam Military Medical University, Hanoi 12108, Vietnam; 4 Department of Nutrition, Military Hospital 103, Vietnam Military Medical University, Hanoi 12108, Vietnam; 5 Faculty of Public Health, Haiphong University of Medicine and Pharmacy, Haiphong 04212, Vietnam; 6 School of Nutrition and Health Sciences, Taipei Medical University, Taipei 11031, Taiwan; 7 Department of Health Promotion, Faculty of Social and Behavioral Sciences, Hanoi University of Public Health, Hanoi 11910, Vietnam

**Keywords:** biomarkers, body water, extracellular water-to-total body water ratio, dengue, length of hospital stay

## Abstract

Objectives: This study investigated the associations of biochemical and body water distribution parameters with dengue status, as well as their discriminatory ability, among hospitalized adults with febrile illnesses and evaluated whether dynamic changes in body water volumes were associated with length of hospital stay (LOS) in dengue patients. Methods: A prospective observational cohort study was conducted at a tertiary care hospital involving 186 hospitalized adults (age ≥ 18 years) with fever onset ≤ 5 days and suspected dengue. Body water parameters were assessed by bioelectrical impedance analysis (BIA) using the InBody S10 body composition analyzer at admission (T1), defervescence (T2), and discharge (T3) in dengue patients and at admission only in other febrile illness (OFI) cases. Laboratory data and LOS were retrieved from the hospital information system. Linear and logistic regression models were used to examine the associations and interactions. Discriminative performance was assessed using a receiver operating characteristic (ROC) curve analysis. Results: The proportion of dengue cases was 55.9% (n = 104). Higher levels of lymphocytes, hematocrit, hemoglobin, AST, and ALT were associated with an increased likelihood of dengue, whereas elevated WBC counts, neutrophils, platelets, CRP, sodium, chloride, and the extracellular water-to-total body water ratio (ECW/TBW) were associated with a reduced likelihood of dengue. ROC analysis indicated that WBC showed the best diagnostic performance. In dengue patients, a greater increase in ECW volume from admission to defervescence was associated with a longer LOS in males, and ratio-based body water parameters showed longitudinal variation across dengue phases. Conclusions: Several hematologic, biochemical, and BIA-derived body water parameters were associated with dengue status. Among dengue patients, dynamic ECW changes were associated with longer LOS in males, and ratio-based fluid indices were more sensitive than absolute water volumes in reflecting fluid redistribution throughout the dengue course.

## 1. Introduction

Dengue is a rapidly emerging mosquito-borne viral infection and a major global public health concern [1]. Nearly half of the world’s population lives in areas at risk of dengue transmission, with an estimated 100–400 million infections reported annually across more than 130 countries. The burden is greatest in tropical regions of Asia, the Americas, and the Western Pacific—particularly in resource-limited settings—where dengue remains a leading cause of morbidity, mortality, and economic disruption [1,2,3]. Early clinical manifestations of dengue are often non-specific and overlap substantially with other febrile illnesses (OFIs) such as malaria, influenza, and leptospirosis—particularly during the initial febrile phase—making timely diagnosis difficult [4]. Although molecular diagnostics, such as RT-PCR, offer high sensitivity and specificity, their use in low- and middle-income countries (LMICs) remains limited due to cost, technical requirements, and restricted laboratory access [5,6]. When available, rapid diagnostic tests have demonstrated variable performance and often limited sensitivity [7].

Early recognition of dengue is essential for prompt clinical management and appropriate triage, and previous studies have identified several useful demographic, clinical, and laboratory indices for distinguishing dengue from OFIs [8,9,10]. Additionally, while most dengue infections are self-limiting, a considerable number of patients require hospitalization each year [11]. During large-scale or prolonged outbreaks, especially in resource-constrained settings, this surge in hospital admissions can significantly strain healthcare services and increase the overall economic burden [12]. Although the length of hospital stay (LOS) for dengue is relatively short [1], it remains an important outcome for patient management and healthcare resource planning and warrants further investigation.

Bioelectrical impedance analysis (BIA) has gained recognition as a noninvasive tool for assessing body water, showing good agreement with reference methods [13,14]. Fluid distribution imbalances measured by BIA have been associated with outcomes in infectious diseases such as HIV, hepatitis, sepsis, and COVID-19 [15,16,17,18]. In dengue, fluid redistribution is a key pathophysiological feature, and BIA offers a practical means to monitor dynamic shifts between intracellular and extracellular compartments [18,19,20,21]. For instance, a shift in fluid from the intracellular to the extracellular compartment during the progression from the acute to the convalescent phase was reported in dengue patients [19]. Similarly, in pediatric populations, the extracellular-to-intracellular water ratio (ECW/ICW), a proxy for extracellular water-to-total body water ratio (ECW/TBW), has been demonstrated to be increased with disease severity [21].

However, the association between body water distribution and dengue status remains underexplored. Moreover, few studies have applied multi-frequency segmental BIA (MF-SBIA) to dengue patients, and there is a notable lack of evidence on the relationship between longitudinal changes in body water parameters and LOS in this population. To address these gaps, we conducted this study to investigate the associations of biomarkers and BIA-derived body water parameters with dengue status and hospitalization duration, as well as their predictive ability, while also characterizing longitudinal changes in body water distribution across dengue phases.

## 2. Materials and Methods

### 2.1. Study Design and Population

A prospective observational cohort study was conducted between September 2024 and June 2025 at the Department of Infectious Diseases, Military Hospital 103, Hanoi, Vietnam. Adults aged 18 years or older presenting with a fever (≥38 °C) at admission and a history of fever lasting fewer than 5 days, along with clinical features suggestive of dengue infection, were considered for enrollment. All patients were managed and monitored daily until hospital discharge at the discretion of clinical staff who were not involved in the study. Exclusion criteria included the presence of implantable medical devices (e.g., pacemakers or metal prosthetics), pregnancy, or the appearance of clinical symptoms of severe dengue at admission. Eventually, a total of 186 patients were included in the final analysis.

### 2.2. Data Collection

#### 2.2.1. BIA Parameters

In this study, body composition was assessed using the portable InBody S10 device (Biospace Co., Ltd., Seoul, Korea), operated by a trained researcher. This analyzer has been previously validated and widely applied across diverse populations [16,22,23]. The standardized protocol involved placing four conventional electrodes on the patients in a supine position—two on the ankles and two on the wrists. All BIA procedures were conducted in strict accordance with the manufacturer’s guidelines, with each session taking approximately 10 min.

All body composition parameters were recorded for each measurement. This cohort focused on body water parameters, including total body water (TBW), extracellular water (ECW), and intracellular water (ICW), as well as the following ratios: ECW/TBW, ICW/TBW, and ECW/ICW. The ECW/TBW ratio was calculated as ECW divided by TBW, expressed as a percentage. Similarly, the ECW/ICW and ICW/TBW ratios were calculated as percentages by dividing ECW by ICW and ICW by TBW, respectively. In addition to whole-body values, segmental body water data were collected from five regions (right arm, left arm, trunk, right leg, and left leg) for further analysis.

#### 2.2.2. Demographic, Clinical, and Laboratory Parameters

The chart review forms (CRFs) were completed by trained research assistants using information extracted from patients’ electronic medical records. These forms captured patients’ demographic characteristics and clinical data, including admission date, discharge date, final diagnosis during hospitalization, comorbidities, and laboratory profiles.

Demographic variables included age and sex (male and female), with age stratified into younger and older adults using a 60-year cut-off based on prior literature [24]. Body weight and height were measured directly by the research team. Body mass index (BMI, kg/m^2^) was calculated by dividing weight (kg) by the square of the height (m^2^). A BMI ≥ 25 was used to classify obesity [25]. The presence of comorbidities such as cardiovascular disease, chronic pulmonary disease, diabetes mellitus, liver disease, and chronic kidney disease was also recorded.

Biological indicators were obtained from routine blood tests at admission and included hematological results, urea, creatinine, liver enzymes (AST, aspartate aminotransferase; ALT, alanine aminotransferase), C-reactive protein (CRP), and electrolyte indices (potassium, sodium, chloride).

The classification of dengue severity adhered strictly to the guidelines for diagnosis and treatment of dengue issued by the WHO and the Ministry of Health of Vietnam, including dengue without warning signs, dengue with warning signs, and severe dengue [4,26].

### 2.3. Study Procedure

On the day of admission, demographic, clinical, and laboratory data were collected from eligible participants, and all anthropometric and BIA tests were conducted by a trained researcher. In our study, patients with OFI underwent a single BIA measurement at admission, while dengue patients were assessed at three specific time points during hospitalization, including the day of admission (T1), the day of defervescence (T2), and the day of discharge (T3), corresponding to the febrile, critical, and recovery phases of dengue illness, respectively. T2 was marked as the day when body temperature decreased and stabilized below 38–37.5 °C, typically occurring around illness days 3–7 [4].

BIA measurements at admission (T1) were conducted prior to the initiation of any medical treatment at the hospital. Similarly, assessments in dengue patients at T2 and T3 time points were performed in the morning, before the initiation of fluid therapy, by the same trained researcher to ensure consistency across repeated measurements. On the day of discharge, data were collected regarding the admission date, discharge date, and final diagnosis during hospitalization.

### 2.4. Study Outcomes

Dengue status was a primary outcome in this cohort, determined by clinicians based on a comprehensive review of clinical and laboratory findings. At Military Hospital 103, patients were identified as dengue cases when they had laboratory-confirmed results with a positive NS1 antigen and/or dengue-specific IgM antibody rapid test [26]. Those with negative results for both NS1 antigen and IgM antibody and without a confirmed alternative diagnosis were categorized as the non-dengue group or as having OFI. Because some patients were hospitalized within the first 1–2 days of illness, initial NS1 and IgM results could be negative despite evolving dengue infection. In such cases, when dengue remained clinically suspected, repeat NS1 antigen and/or dengue IgM testing was conducted during hospitalization by the treating physician. Those with subsequent positive results were classified as dengue and followed according to the study protocol. Patients with persistently negative available tests and no evidence supporting dengue were classified as OFI. LOS was the main outcome for the specific dengue group, calculated as the number of days from admission to discharge.

### 2.5. Statistical Analysis

Categorical variables were summarized as frequencies and percentages, while continuous variables were reported as means with standard deviations (SD) or as medians with interquartile ranges (IQR) for normally and non-normally distributed data, respectively. Group differences were assessed using the Chi-square test or Fisher’s exact test for categorical variables and the independent samples t-test or one-way ANOVA for normally distributed continuous variables. For continuous variables that did not follow a normal distribution, the Mann–Whitney U test was applied.

We used logistic regression models to examine associations between independent parameters, including demographic data, comorbidities, BMI, laboratory, and body composition parameters, and dengue status. Linear regression analyses were conducted to evaluate early predictors of prolonged LOS among dengue patients. We included changes in body water volumes from admission to defervescence, specifically ICW (ΔICW), ECW (ΔECW), TBW (ΔTBW), and the total ECW/TBW ratio (ΔECW/TBW total) in the bivariate linear regression model to predict the LOS. In the multivariate regression analysis, each biomarker or BIA-derived parameter was analyzed in a separate adjusted model, including age, sex, comorbidity, and BMI. This approach was used to reduce model instability and minimize potential multicollinearity. Nevertheless, given the number of parameters examined, our findings should be interpreted cautiously. In this study, we further assessed interactions between laboratory markers and body water parameters in relation to dengue status and LOS. Due to the number of body water variables examined, we focused on two commonly used indices—ECW/TBW and Phase Angle (PhA)—for interaction analyses. Additionally, previous evidence suggests that age and gender may influence fluid volumes [20,27]; therefore, the subgroup analysis was performed by stratifying by sex (male vs. female) and by age (younger adults vs. older adults). A receiver operating characteristic (ROC) curve analysis was utilized to evaluate the diagnostic performance and optimal thresholds of biomarkers and body water parameters in differentiating dengue from OFIs. The cut-offs were determined based on the maximum Youden index.

For longitudinal analyses, a mixed between–within-subjects analysis of variance (ANOVA) with Bonferroni’s multiple comparisons test was conducted to examine the effects of time, age, and their interaction on changes in fluid distribution over the course of dengue. The outcomes of interest for repeated measures included ECW, ICW, TBW, ECW/ICW, ICW/TBW, and ECW/TBW, both in total and at the segmental level. Significant differences were defined as *p* values less than 0.05. All statistical analyses and data visualizations were performed using SPSS 27.0 (SPSS software, SPSS Inc., Armonk, NY, USA).

## 3. Results

### 3.1. Characteristics of the Study Population

A total of 186 patients were included in the study, comprising 104 (55.9%) dengue-confirmed cases. Among them, 50 (26.9%) were elderly (≥60 years), 91 (48.9%) were men, and 60 (32.3%) had at least one comorbidity. There were 33 (17.7%) patients with obesity. The mean LOS was 5.2 ± 2.4 days (Table 1).

Dengue patients exhibited markedly lower WBC counts, neutrophil percentages, and platelet counts. In contrast, the lymphocyte percentage, hematocrit values, and hemoglobin levels were significantly higher in the dengue group (*p* < 0.05). Liver enzyme levels (AST, ALT) were notably elevated in dengue cases. Levels of serum urea, CRP, sodium, and chloride were significantly reduced in dengue patients (*p* < 0.05) (Table 1). Values of creatinine and potassium did not differ significantly between the two groups (*p* > 0.05) (Table 1).

At hospital admission, dengue patients had significantly decreased ECW/ICW and ECW/TBW in total ratios, with consistent trends of ECW/TBW across all body segments (*p* < 0.05). Conversely, the dengue group had significantly higher values in ICW/TBW and PhA (*p* < 0.001; Table 1). Among dengue patients, 65 (62.5%) were diagnosed with dengue without warning signs, while 39 (37.5%) had warning signs; no severe cases were observed in this cohort (Table 1).

### 3.2. Associations of Biomarkers and BIA Parameters with Dengue Status

Multivariate logistic regression results indicated that, among all subjects, increases in lymphocyte percentage, hematocrit, and hemoglobin levels were associated with a higher likelihood of dengue status, with aORs of 1.091 (95% CI = 1.055, 1.127, *p* < 0.001) for lymphocyte percentage, 1.239 (95% CI = 1.128, 1.361, *p* < 0.001) for hematocrit, and 1.052 (95% CI = 1.025, 1.079, *p* < 0.001) for hemoglobin. In contrast, higher levels of WBC count (aOR = 0.595, 95% CI = 0.510, 0.694, *p* < 0.001), neutrophil percentage (aOR = 0.922, 95% CI = 0.898, 0.947, *p* < 0.001), and platelet count (aOR = 0.974, 95% CI = 0.967, 0.982, *p* < 0.001) were associated with a reduced likelihood of dengue. Additionally, negative associations with dengue status were observed in patients with increased levels of CRP (aOR = 0.958, 95% CI = 0.938, 0.979, *p* < 0.001), sodium (aOR = 0.824, 95% CI = 0.736, 0.922, *p* < 0.001), and chloride (aOR = 0.836, 95% CI = 0.756, 0.924, *p* < 0.001), while elevations in AST (aOR = 1.008, 95% CI = 1.003, 1.015, *p* = 0.002) and ALT (aOR = 1.005, 95% CI = 1.001, 1.009, *p* = 0.037) were correlated with an increased likelihood of dengue (Table 2). Subgroup analyses for biomarkers stratified by gender and age are also presented in Table 2 and Table S2.

Among the BIA parameters, higher values of VFA, ECW/ICW, and ECW/TBW were associated with a lower likelihood of dengue (aOR = 0.986, 95% CI = 0.979, 0.998, *p* = 0.019; aOR = 0.825, 95% CI = 0.730, 0.933, *p* = 0.002; and aOR = 0.642, 95% CI = 0.453, 0.860, *p* = 0.004, respectively). Conversely, increased levels of ICW/TBW and PhA were associated with a higher likelihood of dengue (aOR = 1.643, 95% CI = 1.195, 2.259, *p* = 0.030 for ICW/TBW; aOR = 1.800, 95% CI = 1.219, 2.657, *p* = 0.003 for PhA). A higher PBF was associated with lower odds of dengue in univariate analysis (OR = 0.967, 95% CI = 0.937, 0.998, *p* = 0.041) (Table S1), but this association was not statistically significant in the multivariate model (*p* = 0.160) (Table 2). Notably, the associations between BIA parameters (VFA, ECW/ICW, ICW/TBW, ECW/TBW, and PhA) and dengue status were no longer significant in male participants or younger adults (*p* > 0.05) (Table 2 and Table S2).

No significant interactions were identified between laboratory and body water parameters in relation to dengue status (all interaction *p*-values > 0.05) (Table S3).

### 3.3. Discriminative Performance of Biomarkers and BIA Parameters for Differentiating Dengue from OFIs

ROC analysis was performed to evaluate the discriminative performance of biomarkers and BIA parameters for distinguishing dengue (Table 3). WBC demonstrated the highest diagnostic performance across all groups, with AUCs of 0.899 in the overall sample, 0.882 in males, and 0.913 in females, with the highest Youden index observed in females at 0.736 vs. 0.568 in males and 0.657 overall. Platelets consistently showed high performance (AUC: 0.867 overall, 0.844 in males, 0.886 in females), with higher specificity in males (95.3%) and higher Youden index in females (0.691). AST showed comparable performance across groups (AUC: 0.791 overall, 0.802 in males, 0.775 in females), while ALT had lower AUCs (0.693 overall, 0.720 in males, 0.663 in females). CRP demonstrated good discrimination, with slightly higher AUC in females (0.782) compared to males (0.697) and overall (0.741). Acceptable performance was observed in sodium and chloride across groups, with AUCs ranging from 0.644 to 0.706, slightly higher in females. Similarly, ECW/TBW and PhA showed acceptable performance overall but better in females. Particularly, ECW/TBW had AUCs of 0.640 overall, 0.563 in males, and 0.712 in females, while PhA showed AUCs of 0.644 overall, 0.550 in males, and 0.747 in females (Table 3).

The optimal thresholds, along with corresponding sensitivity, specificity, and Youden index in the overall population, men, and women, are reported in Table 3.

### 3.4. Associations of Biomarkers and BIA Parameters with LOS in Dengue Patients

Results of univariate regression analyses are presented in Table S4. Multiple linear regression analyses indicated that a higher WBC count was significantly associated with a longer LOS in the overall sample (B = 0.247, 95% CI = 0.105, 0.390, *p* < 0.001) and in males (B = 0.413, 95% CI = 0.176, 0.649, *p* = 0.001) but not in females (*p* = 0.071). An increase in neutrophils was correlated with prolonged LOS across the total subjects (B = 0.045, 95% CI = 0.023, 0.067, *p* < 0.001), in men (B = 0.045, 95% CI = 0.007, 0.083, *p* = 0.023), and in women (B = 0.040, 95% CI = 0.012, 0.067, *p* = 0.005) (Table 4). Conversely, lymphocytes demonstrated a negative correlation with longer hospital stays in the overall subjects (B = −0.064, 95% CI = −0.091, −0.036, *p* < 0.001), in males (B = −0.086, 95% CI = −0.143, −0.030, *p* = 0.004), and in females (B = −0.050, 95% CI = −0.081, −0.020, *p* = 0.002). Elevated urea levels were associated with longer hospitalization in the total subjects (B = 0.448, 95% CI = 0.201, 0.696, *p* < 0.001), in male patients (B = 0.679, 95% CI = 0.348, 1.009, *p* < 0.001), and in female patients (B = 0.400, 95% CI = 0.008, 0.791, *p* = 0.046). Increased levels of creatinine were correlated with prolonged LOS only in male patients (B = 0.027, 95% CI = 0.002, 0.052, *p* = 0.034). Sodium was positively associated with prolonged hospital stay in the general population (B = 0.101, 95% CI = 0.012, 0.190, *p* = 0.026). However, when stratified by gender, the relationship between sodium level and LOS was no longer significant in both male and female individuals (*p* > 0.05) (Table 4).

Regarding body water parameters, an increase in ΔECW was significantly correlated with prolonged LOS in male patients (B = 2.585, 95% CI = 0.293, 4.877, *p* = 0.029) but not in the overall cohort or in females (*p* > 0.05) (Table 4). The interactions between laboratory parameters and body water indices on LOS were not found in the dengue patients (interaction *p*-values > 0.05) (Table S5).

### 3.5. Changes in Body Water Parameters in Dengue Patients

**Figure 1:**
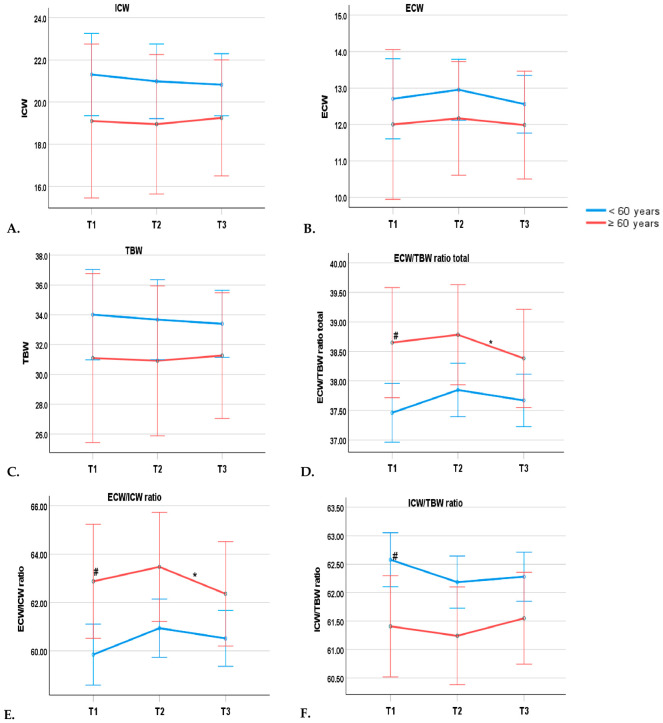
Trends of ICW (**A**), ECW (**B**), TBW (**C**), ECW/TBW (**D**), ECW/ICW (**E**), and ICW/TBW (**F**) parameters in 3 different time points of dengue patients. Data are presented as mean and SEM. # *p* < 0.05 between groups; * *p* < 0.05 between 2 time points according to Bonferroni’s multiple comparison test. Abbreviations: T1, day of admission; T2, day of defervescence; T3, day of discharge; ECW, extracellular water; ICW, intracellular water; TBW, total body water; ICW/TBW, intracellular water-to-total body water ratio; ECW/ICW, extracellular water-to-intracellular water ratio; ECW/TBW, extracellular water-to-total body water ratio.

**Figure 2:**
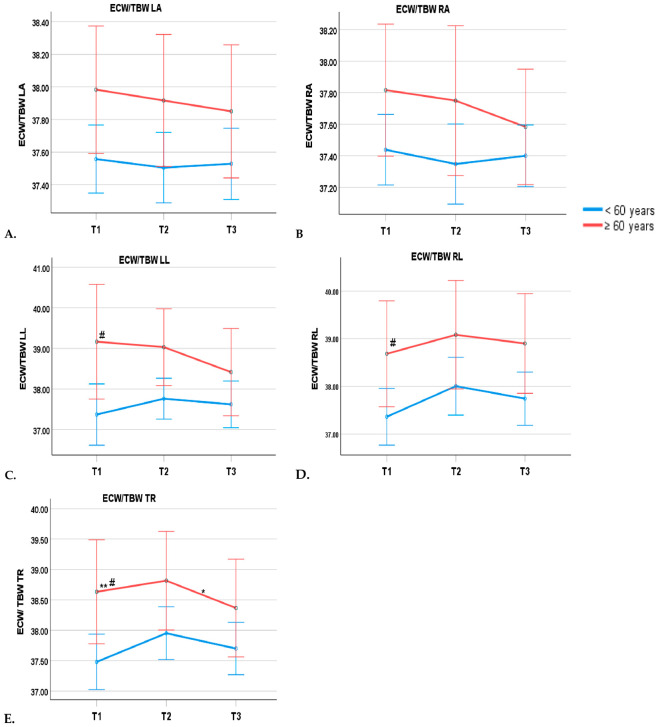
Trends of ECW/TBW in LA (**A**), RA (**B**), LL (**C**), RL (**D**), and TR (**E**) parameters in 3 different time points of dengue patients. Data are presented as mean and SEM. # *p* < 0.05 between groups; ** *p* < 0.05 between times; * *p* < 0.05 between 2 time points according to Bonferroni’s multiple comparison test. Abbreviations: T1, day of admission; T2, day of defervescence; T3, day of discharge; ECW/TBW, extracellular water-to-total body water ratio; RA, right arm; LA, left arm; TR, trunk; RL, right leg; LL, left leg.

In general, absolute body water volumes changed modestly across the clinical course of dengue, whereas ratio-based parameters, particularly ECW/TBW in the trunk, demonstrated more apparent phase-related variation (Figure 1 and Figure 2). In particular, no statistically significant changes were observed across time points or between age groups for ECW, ICW, or TBW (*p* > 0.05) (Table 5, Figure 1A–C). ECW/ICW, ICW/TBW, and ECW/TBW (total) showed significant differences between the two age groups (all *p*-values < 0.05). Specifically, the older group had significantly higher values of ECW/ICW and ECW/TBW (total), while exhibiting a significantly lower ICW/TBW index during the dengue period (Table 5, Figure 1D–F). Notably, Bonferroni’s multiple comparison results revealed significant decreases between the days of defervescence and discharge for ECW/ICW and ECW/TBW total values (*p* < 0.05) (Figure 1D,E).

Segmental analyses indicated that the ECW/TBW ratio values in the lower extremities and trunk were significantly increased in the elderly (*p* < 0.05). Furthermore, a significant time effect was observed in the trunk ECW/TBW, with values decreasing between defervescence and discharge (*p* < 0.05, as determined by Bonferroni’s multiple comparison test) (Table 5, Figure 2C–E). No significant interaction effects between time and age were found among body water parameters (*p* > 0.05) (Table 5).

## 4. Discussion

Our findings demonstrated the associations of laboratory and body water parameters with dengue status, the predictive accuracy of these parameters, and their relationship with LOS in dengue cases. We also identified notable age- and sex-related differences in hydration dynamics, highlighting the importance of interpreting body composition indices within a demographic context. Importantly, longitudinal analyses of body water changes showed that an increase in ΔECW from admission to defervescence was associated with a longer LOS in dengue patients, particularly among males.

Our study illustrated that several biomarkers—including WBC and platelet counts, CRP, and liver enzymes—may serve as useful indicators with good to excellent discriminative performance for distinguishing dengue from OFIs, consistent with findings from previous studies [9,10,28,29]. Our cohort also revealed that sodium and chloride levels may help differentiate dengue from OFIs. Although electrolyte imbalances in dengue patients have been reported in earlier investigations [30,31], the association between electrolyte parameters and dengue status among patients with OFI has been less extensively investigated. Our results highlight the potential value of incorporating early screening for electrolyte indices at admission as part of the routine evaluation of febrile patients in dengue-endemic settings.

Notably, we observed significantly lower CRP levels in the dengue group compared to the OFI group. Furthermore, those with an increased level of CRP were less likely to be diagnosed with dengue, which aligns with several prior reports, where CRP has been explored as a supportive diagnostic indicator to differentiate dengue from other causes of acute fever, such as malaria or leptospirosis [32,33,34,35]. While CRP is not specific for dengue, it may aid in early clinical decision-making, particularly in settings where access to definitive diagnostic tests is limited. A normal CRP level may suggest dengue or another self-limiting viral illness, potentially helping to avoid unnecessary antibiotic use or extensive bacterial work-up, especially relevant in resource-constrained environments or during outbreak situations [36]. However, in some cases, particularly in early dengue progressing toward severe disease, CRP levels may rise and mimic a bacterial infection, which could lead to misclassification [37]. Therefore, CRP measurement should be accompanied by other markers such as total WBC counts, neutrophil counts, platelet trends, and liver enzyme levels to enhance diagnostic accuracy [32], especially when confirmatory testing is pending or unavailable.

Aside from laboratory parameters, our study examined the association between BIA-derived parameters and dengue status. Among body water parameters, the ECW/TBW ratio is widely used to detect abnormal hydration status and serves as a sensitive indicator of fluid imbalance [38]. In our study, a higher ECW/TBW ratio was associated with lower odds of dengue among patients with OFI. Previous research has shown that this ratio can act as a dynamic and prognostic marker in both viral and bacterial infections, particularly in sepsis and bacteremia, where an elevated ECW/TBW level correlates with disease severity, treatment response, and mortality [16,39,40]. A potential physiological explanation for this observation is the release of antidiuretic hormone (ADH) and the presence of hyponatremia—a common electrolyte disturbance in dengue, observed more frequently than in OFIs [41]. Hyponatremia may result from salt depletion, excess free-water retention due to increased metabolism, reduced renal excretion, intracellular sodium influx caused by sodium-potassium pump dysfunction, and renal sodium losses due to acute tubular necrosis [41]. Additionally, inappropriate ADH secretion has been documented in dengue infection, which may further contribute to water retention without proportional sodium retention [42]. This process could lead to a dilutional decrease in serum sodium, promoting a shift of water into cells, increasing the ICW fraction, and relatively reducing ECW, even when TBW is preserved or elevated. This pattern would result in a lower ECW/TBW ratio, consistent with our findings. However, the underlying mechanisms remain uncertain, and our interpretations should be considered speculative.

A novel finding in this study was that a greater increase in ECW volume change (ΔECW) from admission to defervescence was associated with a higher likelihood of prolonged LOS in male dengue patients (B = 2.585, *p* = 0.029). This supports the hypothesis that greater extracellular fluid accumulation—possibly due to persistent endothelial dysfunction or inadequate fluid reabsorption—may contribute to delayed clinical recovery in individuals with dengue. Our result is in line with earlier work, suggesting that relative expansion of ECW during the disease course and into the convalescent stage may serve as a marker for tracking dengue progression [19]. The absence of a significant association in females in our study may be attributable to sex-based physiological differences in fluid distribution, hormonal influences on endothelial integrity, or variations in inflammatory responses [43,44]. In addition, differences in muscle mass and baseline impedance values could influence the accuracy and sensitivity of BIA measurements across sexes [20]. Nevertheless, this finding may reflect either a biologically relevant sex-related difference in fluid dynamics or a chance association that requires further validation.

In examining longitudinal changes in fluid distribution across the three phases of dengue, we observed no statistically significant changes in ECW, ICW, TBW, or their ratios (ECW/ICW, ECW/TBW, ICW/TBW), except for the ECW/TBW ratio in the trunk. These findings align with previous work by Klassen et al., who similarly reported no significant changes in whole-body TBW, ECW, ICW, or ECW/TBW throughout the dengue disease course [19]. We propose that the relative stability of these fluid compartments in non-severe dengue reflects minimal plasma leakage from the vasculature. The extent of fluid shift in such cases is likely too small to produce detectable changes in TBW or extracellular volume. Additionally, minor intravascular fluid losses due to capillary leakage or fever-related dehydration may have been adequately compensated by oral or intravenous (IV) fluid intake during hospitalization, as well as by physiological renal mechanisms that promote water retention [21]. Together, these compensatory responses help maintain whole-body fluid homeostasis and may mask subtle fluid shifts.

Nevertheless, the trunk-specific variation we observed provides an important insight. Segmental BIA measures impedance separately in the limbs and trunk, allowing detection of localized fluid shifts that may be diluted in whole-body averages [45]. Supporting this, Mazariegos et al. reported that trunk impedance showed the weakest correlation with whole-body measurements in dengue patients, suggesting that the trunk may behave differently from the body’s overall average [46]. Our findings may reflect relative redistribution of body water rather than large net changes in total fluid volume, and segmental BIA may offer a more sensitive approach for monitoring fluid redistribution in dengue when available. However, this potential application remains preliminary, and future cohort studies in dengue patients with severe illness or outpatients are needed to validate our findings.

In this study, older dengue patients exhibited significantly higher ECW/TBW ratios in the whole body, trunk, and lower limbs compared to younger adults. These findings align with Akemi Hioka’s study in community-dwelling females, which reported higher values of ECW/TBW in total in elderly individuals compared to non-elderly adults [27]. A prospective U.S. study similarly found that individuals over 61 years of age had lower TBW indices than those aged 21–60 years [47]. This age-related decline in TBW is largely attributable to the loss of skeletal muscle mass—tissue that is rich in water—combined with increased adiposity, which has a lower water content [47].

This study has several limitations. Firstly, it was conducted at a single tertiary care hospital with a relatively small sample size, which may limit the generalizability of the findings and the robustness of the analysis. A multicenter prospective study involving febrile patients, including those with dengue, across different healthcare settings in Vietnam is currently being planned to address this limitation. Secondly, dengue diagnoses were based on routine hospital diagnostic practice using NS1 antigen and/or IgM rapid testing rather than a standardized research diagnostic algorithm. Moreover, RT-PCR confirmation for dengue was not performed in this cohort. As a result, misclassification of early dengue as OFI cannot be excluded, which may have affected internal validity and observed associations. Thirdly, all BIA measurements were performed using the InBody S10 device; results may vary when using other bioimpedance platforms. Fourthly, the study did not include information regarding fluid therapy or other treatments, which may have influenced the results. Fluid administration was reported to alter BIA-derived parameters, particularly ECW, TBW, and ECW/TBW ratio [16,48]. Thus, the serial BIA changes observed during hospitalization in our cohort may reflect both disease-related fluid redistribution and the effects of therapeutic fluid management. In addition, laboratory and BIA results may have been affected by the day of illness at admission or pre-admission hydration status, which was not adjusted for in our analysis. Lastly, although multi-frequency BIA (MF-BIA) shows promise in detecting fluid shifts in dengue, it does not directly measure plasma volume contraction, the pathophysiological hallmark of severe dengue [21]. Therefore, BIA-derived body water parameters should be interpreted as associative findings and considered in conjunction with clinical and laboratory indicators rather than as standalone diagnostic or prognostic measures.

## 5. Conclusions

In this prospective cohort of hospitalized adults with suspected dengue, selected admission hematologic, biochemical, and BIA-derived body water parameters were associated with dengue status. Among the evaluated parameters, WBC exhibited the strongest discriminative performance. In patients with dengue, longitudinal changes were more evident in fluid distribution ratios than in absolute water volumes, with trunk ECW/TBW showing significant variation across disease phases, while greater increases in ECW were associated with longer hospital stays in males. Our findings suggest that BIA-derived fluid indices may provide complementary information on fluid redistribution in dengue, although further prospective studies with larger sample sizes are needed to validate their clinical relevance.

## Figures and Tables

**Table 1:** Characteristics of the study patients.

Parameters	Total (n = 186)	Non-Dengue Patients (n = 82)	Dengue Patients (n = 104)	*p*-Value
Age (n,%)				0.020
<60 years	136 (73.1)	53 (64.6)	83 (79.8)	
≥60 years	50 (26.9)	29 (35.4)	21 (20.2)	
Gender (n, %)				0.461
Male	91 (48.9)	43 (52.4)	48 (46.2)	
Female	95 (51.1)	39 (47.6)	56 (53.8)	
Comorbidity (n, %)				0.003
No	126 (67.7)	46 (56.1)	80 (76.9)	
One or more	60 (32.3)	36 (43.9)	24 (23.1)	
BMI (n, %)				0.416
Non-obese (<25)	153 (82.3)	67 (81.7)	86 (82.7)	
Obese (≥25)	33 (17.7)	15 (18.3)	18 (17.3)	
Length of hospital stay (days)	5.2 ± 2.4	5.6 ± 2.7	4.8 ± 2.1	0.028
Dengue severity (n, %)				
Dengue without warning signs			65 (62.5)	
Dengue with warning signs			39 (37.5)	
**Laboratory parameters at admission**				
WBC (×10^3^/µL)	6.86 ± 4.44	9.88 ± 4.40	4.48 ± 2.72	<0.001
Neutrophils (%)	64.3 ± 19.1	75.5 ± 14.4	55.5 ± 17.8	<0.001
Lymphocytes (%)	21.3 ± 13.9	14.5 ± 10.9	26.6 ± 13.7	<0.001
Hematocrit (%)	41.1 ± 5.0	39.5 ± 4.5	42.3 ± 5.1	<0.001
Hemoglobin (g/L)	136.1 ± 16.7	132.0 ± 14.5	139.4 ± 17.6	0.002
Platelets (×10^3^/µL)	169.5 ± 76.7	220.5 ± 73.4	129.4 ± 51.4	<0.001
AST (IU/L)	49.8 (25.6–96.4)	25.1 (19.7–50.0)	71.2 (43.1–125.5)	<0.001
ALT (IU/L)	34.7 (21.3–69.8)	22.9 (18.0–42.9)	42.5 (27.0–90.6)	<0.001
Urea (mmol/L)	4.6 ± 2.2	5.1 ± 2.6	4.2 ± 1.7	0.011
Creatinine (µmol/L)	89.1 (72.9–106.2)	91.2 (71.0–106.5)	86.9 (74.0–103.6)	0.792
CRP (mg/L)	12.5 (4.6–39.0)	31.7 (9.3–105.7)	8.7 (3.2–16.3)	<0.001
Potassium (mmol/L)	3.60 ± 0.39	3.60 ± 0.30	3.61 ± 0.46	0.970
Sodium (mmol/L)	138.5 ± 4.1	139.5 ± 3.2	137.4 ± 4.7	0.001
Chloride (mmol/L)	103.5 ± 3.8	104.5 ± 3.9	102.5 ± 3.4	0.001
**BIA parameters at admission**				
Protein, kg	8.81 ± 1.82	8.62 ± 1.79	8.95 ± 1.84	0.216
Mineral, kg	3.05 ± 0.79	2.98 ± 0.93	3.10 ± 0.65	0.275
SLM, kg	42.0 ± 8.4	41.3 ± 8.3	42.6 ± 8.5	0.314
FFM, kg	44.6 ± 8.9	43.8 ± 8.7	45.2 ± 9.0	0.298
SMM, kg	24.5 ± 5.5	24.0 ± 5.4	25.0 ± 5.6	0.214
PBF, %	23.5 ± 9.4	25.1 ± 9.9	22.3 ± 8.8	0.039
BCM, kg	29.2 ± 6.1	28.5 ± 6.0	29.6 ± 6.1	0.218
VFA, cm^2^	62.1 ± 27.4	71.5 ± 42.5	54.7 ± 31.1	0.002
SMI, kg/m^2^	6.82 ± 1.53	6.79 ± 1.98	6.85 ± 1.06	0.780
ICW, L	20.4 ± 4.2	19.9 ± 4.2	20.7 ± 4.3	0.214
ECW, L	12.4 ± 2.3	12.3 ± 2.3	12.4 ± 2.3	0.707
TBW, L	32.7 ± 6.5	32.2 ± 6.4	33.1 ± 6.6	0.345
Segmental Water (RA), L	1.79 ± 0.49	1.76 ± 0.47	1.81 ± 0.50	0.447
Segmental Water (LA), L	1.78 ± 0.47	1.73 ± 0.45	1.81 ± 0.49	0.276
Segmental Water (TR), L	15.42 ± 2.94	15.12 ± 2.93	15.66 ± 2.94	0.218
Segmental Water (RL), L	5.16 ± 1.19	4.97 ± 1.21	5.30 ± 1.16	0.062
Segmental Water (LL), L	5.16 ± 1.17	4.99 ± 1.19	5.29 ± 1.14	0.080
ECW/ICW, %	61.1 ± 3.0	62.0 ± 3.2	60.3 ± 2.7	<0.001
ICW/TBW, %	62.1 ± 1.2	61.7 ± 1.2	62.4 ± 1.1	<0.001
ECW/TBW (Total), %	37.9 ± 1.1	38.2 ± 1.2	37.6 ± 1.0	<0.001
ECW/TBW (RA), %	37.7 ± 0.5	37.9 ± 0.6	37.6 ± 0.4	0.002
ECW/TBW (LA), %	37.9 ± 0.5	38.0 ± 0.6	37.8 ± 0.4	0.016
ECW/TBW (TR), %	37.9 ± 1.2	38.2 ± 1.2	37.6 ± 1.0	<0.001
ECW/TBW (RL), %	38.0 ± 1.6	38.5 ± 1.5	37.6 ± 1.5	<0.001
ECW/TBW (LL), %	37.9 ± 1.7	38.3 ± 1.6	37.6 ± 1.7	0.003
PhA (^o^)	5.77 ± 0.97	5.48 ± 1.04	6.00 ± 0.86	<0.001

Categorical variables are presented as n (%). Normally distributed continuous data are demonstrated as mean ± SD. Skewed continuous data are demonstrated as median and interquartile range (IQR). Abbreviations: BMI, Body Mass Index; WBC, white blood cell; AST, aspartate aminotransferase; ALT, alanine aminotransferase; CRP, C-reactive protein; BIA, bioelectrical impedance analysis; SLM, soft lean mass; FFM, fat-free mass; SMM, skeletal muscle mass; PBF, percent body fat; BCM, body cell mass; VFA, visceral fat area; SMI, skeletal muscle mass index; RA, right arm; LA, left arm; TR, trunk; RL, right leg; LL, left leg; ICW, intracellular water; ECW, extracellular water; TBW, total body water; ECW/ICW, extracellular water-to-intracellular water ratio; ICW/TBW, intracellular water-to-total body water ratio; ECW/TBW, extracellular water-to-total body water ratio; PhA, phase angle; ^o^, the unit of Phase angle.

**Table 2:** Associations of biomarkers and BIA parameters with dengue status in the total population, stratified by gender (n = 186).

Parameters			Dengue			
	Overall Sample		Males		Females	
	aOR (95% CI)	*p*-Value	aOR (95% CI)	*p*-Value	aOR (95% CI)	*p*-Value
**Laboratory parameters**						
WBC, 1 × 10^3^/µL increase	0.595 (0.510, 0.694)	<0.001	0.571 (0.449, 0.725)	<0.001	0.605 (0.491, 0.746)	<0.001
Neutrophils, 1% increase	0.922 (0.898, 0.947)	<0.001	0.918 (0.882, 0.954)	<0.001	0.921 (0.888, 0.957)	<0.001
Lymphocytes, 1% increase	1.091 (1.055, 1.127)	<0.001	1.087 (1.037, 1.140)	<0.001	1.099 (1.048, 1.153)	<0.001
Hematocrit, 1% increase	1.239 (1.128, 1.361)	<0.001	1.340 (1.154, 1.557)	<0.001	1.195 (1.045, 1.368)	0.009
Hemoglobin, 1 g/L increase	1.052 (1.025, 1.079)	<0.001	1.075 (1.034, 1.117)	<0.001	1.032 (0.993, 1.073)	0.112
Platelets, 1 × 10^3^/µL increase	0.974 (0.967, 0.982)	<0.001	0.974 (0.963, 0.985)	<0.001	0.974 (0.964, 0.985)	<0.001
AST, 1 IU/L increase	1.008 (1.003, 1.015)	0.002	1.022 (1.009, 1.036)	<0.001	1.005 (1.001, 1.010)	0.051
ALT, 1 IU/L increase	1.005 (1.001, 1.009)	0.037	1.015 (1.003, 1.026)	0.013	1.003 (0.999, 1.006)	0.146
Urea, 1 mmol/L increase	0.842 (0.696, 1.019)	0.077	0.889 (0.702, 1.127)	0.331	0.724 (0.520, 1.007)	0.055
Creatinine, 1 µmol/L increase	1.004 (0.990, 1.019)	0.586	1.008 (0.992, 1.025)	0.331	0.987 (0.955, 1.021)	0.451
CRP, 1 mg/L increase	0.958 (0.938, 0.979)	<0.001	0.952 (0.921, 0.983)	0.003	0.959 (0.923, 0.983)	0.003
Potassium, 1 mmol/L increase	1.204 (0.517, 2.805)	0.667	3.173 (0.828, 12.155)	0.092	0.563 (0.173, 1.832)	0.340
Sodium, 1 mmol/L increase	0.824 (0.736, 0.922)	<0.001	0.826 (0.710, 0.960)	0.013	0.819 (0.691, 0.971)	0.022
Chloride, 1 mmol/L increase	0.836 (0.756, 0.924)	<0.001	0.854 (0.746, 0.976)	0.021	0.817 (0.703, 0.949)	0.008
**BIA parameters**						
Protein, 1 kg increase	1.090 (0.899, 1.322)	0.378	0.713 (0.535, 1.059)	0.112	2.126 (1.357, 3.329)	<0.001
Mineral, 1 kg increase	1.191 (0.794, 1.788)	0.399	0.859 (0.529, 1.394)	0.538	3.742 (1.287, 10.878)	0.015
SLM, 1 kg increase	1.015 (0.973, 1.058)	0.489	0.926 (0.869, 1.086)	0.106	1.156 (1.061, 1.279)	0.001
FFM, 1 kg increase	1.015 (0.976, 1.056)	0.465	0.933 (0.880, 1.089)	0.120	1.151 (1.055, 1.256)	0.002
SMM, 1 kg increase	1.029 (0.966, 1.097)	0.372	0.896 (0.815, 1.098)	0.203	1.280 (1.105, 1.482)	0.001
PBF, 1% increase	0.973 (0.937, 1.011)	0.160	1.021 (0.969, 1.077)	0.439	0.910 (0.856, 0.968)	0.003
BCM, 1 kg increase	1.026 (0.969, 1.088)	0.379	0.904 (0.829, 1.085)	0.102	1.252 (1.095, 1.432)	0.001
VFA, 1 cm^2^ increase	0.986 (0.979, 0.998)	0.019	0.995 (0.982, 1.008)	0.435	0.978 (0.963, 0.993)	0.004
SMI, 1 kg/m^2^ increase	0.984 (0.796, 1.216)	0.879	0.531 (0.310, 1.008)	0.061	2.687 (1.490, 4.845)	0.001
ICW, 1 L increase	1.039 (0.956, 1.128)	0.372	0.866 (0.766, 1.098)	0.123	1.378 (1.138, 1.668)	0.001
ECW, 1 L increase	1.013 (0.870, 1.179)	0.870	0.725 (0.573, 1.016)	0.107	1.595 (1.166, 2.183)	0.004
TBW, 1 L increase	1.018 (0.964, 1.074)	0.181	0.903 (0.833, 1.080)	0.150	1.215 (1.078, 1.370)	0.001
ECW/ICW, 1% increase	0.825 (0.730, 0.933)	0.002	0.933 (0.796, 1.094)	0.396	0.670 (0.529, 0.848)	<0.001
ICW/TBW, 1% increase	1.643 (1.195, 2.259)	0.030	1.188 (0.785, 1.798)	0.416	2.847 (1.536, 5.277)	<0.001
ECW/TBW in total, 1% increase	0.642 (0.453, 0.860)	0.004	0.880 (0.576, 1.344)	0.555	0.356 (0.192, 0.662)	0.001
PhA, 1 degree increase	1.800 (1.219, 2.657)	0.003	1.033 (0.612, 1.744)	0.902	3.801 (1.862, 7.758)	<0.001

Results obtained after adjusting for age, gender, comorbidity, and BMI. Abbreviations: aOR, adjusted odds ratio; 95% CI, 95% confidence interval; WBC, white blood cell; AST, aspartate aminotransferase; ALT, alanine aminotransferase; CRP, C-reactive protein; BIA, bioelectrical impedance analysis; SLM, soft lean mass; FFM, fat-free mass; SMM, skeletal muscle mass; PBF, percent body fat; BCM, body cell mass; VFA, visceral fat area; SMI, skeletal muscle mass index; ICW, intracellular water; ECW, extracellular water; TBW, total body water; ECW/ICW, extracellular water-to-intracellular water ratio; ICW/TBW, intracellular water-to-total body water ratio; ECW/TBW, extracellular water-to-total body water ratio; PhA, phase angle.

**Table 3:** Receiver operating characteristic (ROC) analysis of laboratory and body water parameters for differentiating dengue in overall patients, stratified by gender.

	Cut-Off Value	AUC	95% CI	Sensitivity (%)	Specificity (%)	*p*-Value	Youden Index
Overall sample (n = 186)							
WBC, 10^3^/µL	5.55	0.899	0.854–0.943	77.9	87.8	<0.001	0.657
Platelets, 10^3^/µL	135.5	0.867	0.815–0.918	65.4	92.7	<0.001	0.581
AST, IU/L	37.0	0.791	0.722–0.861	85.0	64.6	<0.001	0.496
ALT, IU/L	24.0	0.693	0.613–0.772	83.7	53.2	<0.001	0.369
CRP, mg/L	26.1	0.741	0.655–0.827	87.2	56.5	<0.001	0.437
Sodium, mmol/L	139.5	0.673	0.590–0.757	75.6	57.5	<0.001	0.331
Chloride, mmol/L	104.8	0.668	0.584–0.752	78.0	51.2	<0.001	0.292
ECW/TBW, %	38.4	0.640	0.560–0.720	53.8	70.7	0.001	0.245
PhA, ^o^	5.65	0.644	0.564–0.724	63.5	57.3	0.001	0.208
Males (n = 91)							
WBC, 10^3^/µL	8.20	0.882	0.813–0.950	91.7	65.1	<0.001	0.568
Platelets, 10^3^/µL	115.0	0.844	0.766–0.922	60.4	95.3	<0.001	0.557
AST, IU/L	38.5	0.802	0.709–0.895	80.9	72.1	<0.001	0.530
ALT, IU/L	34.3	0.720	0.614–0.827	69.6	67.4	<0.001	0.370
CRP, mg/L	30.5	0.697	0.575–0.820	92.1	48.7	0.003	0.408
Sodium, mmol/L	139.7	0.688	0.572–0.804	80.0	62.8	0.003	0.428
Chloride, mmol/L	103.3	0.644	0.525–0.763	65.0	65.1	0.024	0.301
ECW/TBW, %	37.6	0.563	0.443–0.684	60.4	55.8	0.300	0.162
PhA, ^o^	4.55	0.550	0.429–0.672	97.9	23.3	0.408	0.212
Females (n = 95)							
WBC, 10^3^/µL	5.31	0.913	0.854–0.972	83.9	89.7	<0.001	0.736
Platelets, 10^3^/µL	151.5	0.886	0.813–0.960	76.8	92.3	<0.001	0.691
AST, IU/L	26.5	0.775	0.666–0.884	98.1	55.6	<0.001	0.537
ALT, IU/L	22.3	0.663	0.543–0.783	82.7	52.8	0.010	0.355
CRP, mg/L	13.7	0.782	0.659–0.905	77.5	76.7	<0.001	0.542
Sodium, mmol/L	138.6	0.667	0.547–0.787	64.3	64.9	0.011	0.292
Chloride, mmol/L	105.0	0.706	0.589–0.824	83.3	54.1	0.002	0.374
ECW/TBW, %	38.6	0.712	0.610–0.814	89.3	41.0	<0.001	0.303
PhA, ^o^	5.80	0.747	0.651–0.844	51.8	87.2	<0.001	0.390

Abbreviations: AUC, area under the curve; 95%CI, 95% confidence interval; WBC, white blood cell; AST, aspartate aminotransferase; ALT, alanine aminotransferase; CRP, C-reactive protein; ECW/TBW, extracellular water-to-total body water ratio; PhA, phase angle.

**Table 4:** Associations of biomarkers and BIA parameters with length of hospital stay in overall dengue patients, stratified by gender (n = 104).

Parameters	Length of Hospital Stay					
	Overall Sample		Males		Females	
	B (95% CI)	*p*-Value	B (95% CI)	*p*-Value	B (95% CI)	*p*-Value
**Laboratory parameters**						
WBC, 1 × 10^3^/µL increase	0.247 (0.105, 0.390)	<0.001	0.413 (0.176, 0.649)	0.001	0.168 (−0.015, 0.351)	0.071
Neutrophils, 1% increase	0.045 (0.023, 0.067)	<0.001	0.045 (0.007, 0.083)	0.023	0.040 (0.012, 0.067)	0.005
Lymphocytes, 1% increase	−0.064 (−0.091, −0.036)	<0.001	−0.086 (−0.143, −0.030)	0.004	−0.050 (−0.081, −0.020)	0.002
Hematocrit, 1% increase	0.001 (−0.102, 0.103)	0.989	−0.024 (−0.189, 0.142)	0.775	0.034 (−0.104, 0.172)	0.620
Hemoglobin, 1 g/L increase	−0.013 (−0.044, 0.017)	0.393	−0.011 (−0.059, 0.036)	0.636	−0.012 (−0.055, 0.031)	0.578
Platelets, 1 × 10^3^/µL increase	0.013 (−0.005, 0.020)	0.054	0.022 (−0.010, 0.033)	0.068	0.004 (−0.005, 0.014)	0.373
AST, 1 IU/L increase	−0.001 (−0.003, 0.002)	0.640	−0.003 (−0.006, 0.001)	0.173	0.001 (−0.002, 0.004)	0.357
ALT, 1 IU/L increase	0.001 (−0.003, 0.003)	0.801	−0.005 (−0.014, 0.005)	0.326	0.001 (−0.002, 0.004)	0.504
Urea, 1 mmol/L increase	0.448 (0.201, 0.696)	<0.001	0.679 (0.348, 1.009)	<0.001	0.400 (0.008, 0.791)	0.046
Creatinine, 1 µmol/L increase	0.016 (−0.001, 0.033)	0.069	0.027 (0.002, 0.052)	0.034	0.025 (−0.025, 0.075)	0.316
CRP, 1 mg/L increase	0.024 (−0.017, 0.066)	0.250	0.023 (−0.054, 0.101)	0.548	0.033 (−0.013, 0.078)	0.154
Potassium,1 mmol/L increase	−0.641 (−1.595, 0.314)	0.185	−1.232 (−2.912, 0.448)	0.145	−0.110 (−1.299, 1.078)	0.852
Sodium, 1 mmol/L increase	0.101 (0.012, 0.190)	0.026	0.098 (−0.015, 0.211)	0.088	0.038 (−0.150, 0.226)	0.687
Chloride, 1 mmol/L increase	0.058 (−0.071, 0.187)	0.375	0.128 (−0.071, 0.327)	0.199	0.008 (−0.165, 0.182)	0.923
**BIA parameters**						
Protein, 1 kg increase	0.052 (−0.175, 0.268)	0.862	−0.044 (−0.376, 0.296)	0.643	0.108 (−0.215, 0.250)	0.435
Mineral, 1 kg increase	0.161 (−0.492, 0.761)	0.569	0.094 (−0.786, 0.863)	0.836	0.203 (−0.804, 1.037)	0.461
SLM, 1 kg increase	0.015 (−0.040, 0.056)	0.635	−0.012 (−0.078, 0.056)	0.739	0.028 (−0.048, 0.099)	0.352
FFM, 1 kg increase	0.021 (−0.045, 0.066)	0.730	−0.009 (−0.078, 0.071)	0.780	0.041 (−0.049, 0.110)	0.572
SMM, 1 kg increase	0.019 (−0.069, 0.099)	0.879	−0.018 (−0.138, 0.097)	0.785	0.043 (−0.086, 0.152)	0.563
PBF, 1% increase	0.016 (−0.045, 0.068)	0.659	−0.009 (−0.079, 0.084)	0.771	0.047 (−0.042, 0.201)	0.403
BCM, 1 kg increase	0.018 (−0.059, 0.088)	0.769	−0.025 (−0.137, 0.095)	0.687	0.046 (−0.068, 0.128)	0.687
VFA, 1 cm^2^ increase	0.009 (−0.008, 0.025)	0.360	0.003 (−0.010, 0.020)	0.914	0.019 (−0.008, 0.042)	0.322
SMI, 1 kg/m^2^ increase	0.176 (−0.245, 0.650)	0.509	−0.168 (−0.861, 0.497)	0.648	0.392 (−0.147, 0.912)	0.263
ICW, 1 L increase	0.028 (−0.086, 0.316)	0.585	−0.032 (−0.174, 0.202)	0.591	0.061 (−0.098, 0.102)	0.446
ECW, 1 L increase	0.065 (−0.138, 0.354)	0.675	−0.019 (−0.298, 0.366)	0.941	0.098 (−0.168, 0.462)	0.489
TBW, 1 L increase	0.025 (−0.059, 0.097)	0.561	−0.019 (−0.214, 0.103)	0.810	0.041 (−0.067, 0.205)	0.540
ECW/ICW, 1% increase	0.042 (−0.125, 0.211)	0.545	0.167 (−0.071, 0.402)	0.213	−0.085 (−0.268, 0.215)	0.501
ICW/TBW, 1% increase	−0.099 (−0.474, 0.405)	0.570	−0.413 (−1.104, 0.316)	0.098	0.189 (−0.386, 0.960)	0.548
ECW/TBW in total, 1% increase	0.098 (−0.408, 0.556)	0.769	0.414 (−0.324, 0.989)	0.346	−0.172 (−0.781, 0.482)	0.486
PhA, 1 degree increase	0.033 (−0.552, 0.600)	0.895	−0.298 (−1.189, 0.536)	0.326	0.362 (−0.413, 0.939)	0.320
ΔICW, 1 L increase	0.502 (−0.820, 1.714)	0.318	1.502 (−0.205, 3.108)	0.079	−0.926 (−2.910, 0.986)	0.308
ΔECW, 1 L increase	0.105 (−1.218, 1.429)	0.874	2.585 (0.293, 4.877)	0.029	−1.204 (−2.737, 0.330)	0.118
ΔTBW, 1 L increase	−0.057 (−0.734, 0.620)	0.886	0.857 (−0.235, 1.950)	0.118	−0.831 (−1.709, 0.048)	0.063
ΔECW/TBW total, 1% increase	0.356 (−0.559, 1.287)	0.355	0.049 (−2.845, 3.302)	0.916	0.501 (−0.767, 1.786)	0.325

Results obtained after adjusting for age, gender, BMI, and comorbidity. Abbreviations: B, regression coefficients; 95% CI, 95% confidence interval; WBC, white blood cell; AST, aspartate aminotransferase; ALT, alanine aminotransferase; CRP, C-reactive protein; BIA, bioelectrical impedance analysis; SLM, soft lean mass; FFM, fat-free mass; SMM, skeletal muscle mass; PBF, percent body fat; BCM, body cell mass; VFA, visceral fat area; SMI, skeletal muscle mass index; ICW, intracellular water; ECW, extracellular water; TBW, total body water; ECW/ICW, extracellular water-to-intracellular water ratio; ICW/TBW, intracellular water-to-total body water ratio; ECW/TBW, extracellular water-to-total body water ratio; PhA, phase angle; ΔICW, the difference in ICW values between the day of admission and defervescence; ΔECW, the difference in ECW values between the day of admission and defervescence; ΔTBW, the difference in TBW values between the day of admission and defervescence; ΔECW/TBW total, the difference in ECW/TBW total values between the day of admission and defervescence.

**Table 5:** The results of the mixed between–within-subjects ANOVA for body water parameters in dengue patients.

Parameters	Time Effect			Interaction Effect (Time and Age)			Age Effect		
	F-Value	*p*-Value	Eta Squared	F-Value	*p*-Value	Eta Squared	F-Value	*p*-Value	Eta Squared
ECW	0.195	0.795	0.008	0.126	0.857	0.005	0.476	0.497	0.019
ICW	0.431	0.547	0.017	0.776	0.399	0.030	1.201	0.284	0.046
TBW	0.241	0.688	0.010	0.524	0.520	0.021	0.917	0.348	0.035
ICW/TBW	1.341	0.270	0.051	0.772	0.458	0.030	4.690	0.040	0.158
ECW/ICW	2.393	0.112	0.087	0.963	0.376	0.037	4.684	0.040	0.158
ECW/TBW, total	1.877	0.164	0.070	1.057	0.335	0.041	4.597	0.042	0.155
ECW/TBW (RA)	1.438	0.247	0.054	1.118	0.335	0.043	2.117	0.158	0.078
ECW/TBW (LA)	0.367	0.694	0.014	0.168	0.844	0.007	3.826	0.062	0.133
ECW/TBW (TR)	4.067	0.048	0.141	1.459	0.243	0.055	4.191	0.046	0.144
ECW/TBW (RL)	2.563	0.117	0.093	0.144	0.740	0.006	4.436	0.045	0.151
ECW/TBW (LL)	0.975	0.356	0.038	1.665	0.208	0.062	4.985	0.035	0.166

Abbreviations: ECW, extracellular water; ICW, intracellular water; TBW, total body water; ICW/TBW, intracellular water-to-total body water ratio; ECW/ICW, extracellular water-to-intracellular water ratio; ECW/TBW, extracellular water-to-total body water ratio; RA, right arm; LA, left arm; TR, trunk; RL, right leg; LL, left leg.

## Data Availability

The data supporting the findings of this study can be obtained from the corresponding author upon reasonable request.

## Supplementary Materials

The following supporting information can be downloaded at https://www.mdpi.com/article/10.3390/pathogens15050501/s1, Table S1: Factors associated with dengue status in OFI patients from bivariate regression analysis (n = 186), Table S2: Associations of biomarkers and BIA parameters with dengue status, stratified by age, in multivariable regression analysis, Table S3: Interactions of biomarkers, ECW/TBW, and PhA on dengue in the study population (n = 186), Table S4: Factors associated with length of hospital stay in dengue patients from bivariate regression analysis (n = 104), Table S5: Interactions of biomarkers, ECW/TBW, and PhA on LOS in dengue patients (n = 104)

## Abbreviations

BCM	Body Cell Mass
BMI	Body Mass Index
BIA	Bioelectrical Impedance Analysis
ECW	Extracellular Water
FFM	Fat-free Mass
ICW	Intracellular Water
MF-BIA	Multi-frequency Bioelectrical Impedance Analysis
PhA	Phase Angle
PBF	Percent Body Fat
RT-PCR	Reverse Transcription Polymerase Chain Reaction
SLM	Soft Lean Mass
SMM	Skeletal Muscle Mass
SMI	Skeletal Muscle Mass Index
TBW	Total Body Water
VFA	Visceral Fat Area
