# Supplementary material for: Associations of Biomarkers and Body Water with Dengue Status and Length of Hospital Stay: A Single-Center Observational Study
Source: Pathogens. 2026 May 6;15(5):501. doi: 10.3390/pathogens15050501 (PMC13210188; doi:10.3390/pathogens15050501)
Supplement: Supplementary file 1 [file pathogens-15-00501-s001.zip › pathogens-4216585-supplementary.pdf]

Table S1: Factors associated with dengue status in OFI patients from bivariate regression analysis (n=186)

Parameters	Dengue	
	Total population	
	OR (95% CI)	p-value
Age		
< 60 years	Ref	
≥ 60 years	0.462 (0.239,0.894)	0.022
Gender		
Male	Ref	
Female	1.286 (0.720,2.298)	0.395
Comorbidity		
No	Ref	
One or more	0.383 (0.204,0.721)	0.003
BMI		
Non-obese (< 25)	Ref	
Obese (≥ 25)	0.946 (0.444,2.015)	0.885
**Laboratory parameters**		
WBC, $1\times10^3/\mu L$ increase	0.594 (0.511,0.690)	<0.001
Neutrophils, 1 % increase	0.926 (0.904,0.949)	<0.001
Lymphocytes, 1 % increase	1.090 (1.057,1.125)	<0.001
Hematocrit, 1 % increase	1.129 (1.056,1.206)	<0.001
Hemoglobin, 1 g/L increase	1.029 (1.010,1.048)	0.003
Platelets, $1\times10^3/\mu L$ increase	0.975 (0.968,0.982)	<0.001
AST, 1 IU/L increase	1.009 (1.004,1.015)	0.001
ALT, 1 IU/L increase	1.005 (1.001,1.009)	0.046
Urea, 1 mmol/L increase	0.806 (0.677,0.959)	0.015
Creatinine, 1 µmol/L increase	0.999 (0.987,1.011)	0.895
CRP, 1 mmol/L increase	0.959 (0.940,0.978)	<0.001
Potassium, 1 mmol/L increase	1.015 (0.461,2.236)	0.970
Sodium, 1 mmol/L increase	0.833 (0.746,0.929)	0.001
Chloride, 1 mmol/L increase	0.856 (0.780,0.940)	0.001
**BIA parameters**		
Protein, 1 kg increase	1.107 (0.942,1.301)	0.215
Mineral, 1 kg increase	1.237 (0.838,1.828)	0.284
SLM, 1 kg increase	1.018 (0.983,1.054)	0.312
FFM, 1 kg increase	1.018 (0.985,1.052)	0.297
SMM, 1 kg increase	1.034 (0.981,1.091)	0.214
PBF, 1 percent increase	0.967 (0.937,0.998)	0.041
BCM, 1 kg increase	1.031 (0.982,1.082)	0.217
VFA, 1 cm^2 increase	0.988 (0.979,0.996)	0.003
SMI, 1 kg/m^2 increase	1.028 (0.847,1.248)	0.780
ICW, 1 L increase	1.045 (0.975,1.120)	0.213
ECW, 1 L increase	1.025 (0.902,1.164)	0.706
TBW, 1 L increase	1.002 (0.977,1.069)	0.344
ECW/ICW, 1 % increase	0.811 (0.727,0.906)	<0.001
ICW/TBW, 1 % increase	1.718 (1.291,2.287)	<0.001
ECW/TBW in total, 1 % increase	0.593 (0.444,0.791)	<0.001
PhA, 1 degree increase	1.792 (1.293,2.483)	<0.001

Abbreviations: OR, odds ratio; 95% CI, 95% confidence interval; Ref, reference; WBC, white blood cell; AST, aspartate aminotransferase; ALT, alanine aminotransferase; CRP, C–reactive protein; BIA, bioelectrical impedance analysis; SLM, soft lean mass; FFM, fat free mass; SMM, skeletal muscle mass; PBF, percent body fat; BCM, body cell mass; VFA, visceral fat area; SMI, skeletal muscle mass index; ICW, intracellular water; ECW, extracellular water; TBW, total body water; ECW/ICW, extracellular

water–to–intracellular water ratio; ICW/TBW, intracellular water–to–total body water ratio; ECW/TBW, extracellular water–to–total body water ratio; PhA, phase angle.

Table S2: Associations of biomarkers and BIA parameters with dengue, stratified by age, in multivariable regression analysis.

Parameters	Dengue			
	Younger adults (< 60 years)		Older adults (≥ 60 years)	
	aOR (95% CI)	p-value	aOR (95% CI)	p-value
**Laboratory parameters**				
WBC, $1\times10^3/\mu L$ increase	0.552 (0.454,0.672)	<0.001	0.702 (0.547,0.900)	0.005
Neutrophils, 1 % increase	0.917 (0.887,0.948)	<0.001	0.926 (0.886,0.969	<0.001
Lymphocytes, 1 % increase	1.095 (1.051,1.140)	<0.001	1.091 (1.028,1.157)	0.004
Hematocrit, 1 % increase	1.222 (1.091,1.369)	<0.001	1.378 (1.119,1.696)	0.003
Hemoglobin, 1 g/L increase	1.043 (1.012,1.075)	0.007	1.095 (1.030,1.165)	0.004
Platelets, $1\times10^3/\mu L$ increase	0.978 (0.970,0.986)	<0.001	0.952 (0.926,0.977)	<0.001
AST, 1 IU/L increase	1.006 (0.999,1.011)	0.052	1.023 (1.006,1.039)	0.007
ALT, 1 IU/L increase	1.002 (0.998,1.006)	0.268	1.025 (1.005,1.044)	0.012
Urea, 1 mmol/L increase	0.882 (0.696,1.118)	0.882	0.776 (0.551,1.095)	0.149
Creatinine, 1 μmol/L increase	1.004 (0.985,1.024)	0.661	1.005 (0.982,1.029)	0.658
CRP, 1 mmol/L increase	0.966 (0.946,0.985)	<0.001	0.926 (0.865,0.992)	0.029
Potassium, 1 mmol/L increase	0.991 (0.350,2.805)	0.987	1.852 (0.409,8.401)	0.424
Sodium, 1 mmol/L increase	0.844 (0.734,0.972)	0.018	0.783 (0.645,0.951)	0.013
Chloride, 1 mmol/L increase	0.848 (0.747,0.962)	0.011	0.817 (0.689,0.969)	0.020
**BIA parameters**				
Protein, 1 kg increase	1.028 (0.828,1.275)	0.805	1.274 (0.837,1.937)	0.258
Mineral, 1 kg increase	1.125 (0.739,1.713)	0.581	1.350 (0.431,4.224)	0.606
SLM, 1 kg increase	1.005 (0.959,1.053)	0.848	1.037 (0.951,1.132)	0.410
FFM, 1 kg increase	1.005 (0.962,1.051)	0.812	1.034 (0.953,1.123)	0.421
SMM, 1 kg increase	1.009 (0.940,1.084)	0.801	1.083 (0.943,1.243)	0.257
PBF, 1 % increase	0.988 (0.946,1.032)	0.593	0.916 (0.848,0.990)	0.027
BCM, 1 kg increase	1.008 (0.944,1.076)	0.810	1.075 (0.948,1.219)	0.259
VFA, 1 cm^2 increase	0.993 (0.982,1.004)	0.235	0.966 (0.942,0.990)	0.006
SMI, 1 kg/m^2 increase	1.427 (0.730,2.789)	0.298	1.427 (0.730,2.789)	0.298
ICW, 1 L increase	1.012 (0.922,1.110)	0.806	1.111 (0.928,1.331)	0.251
ECW, 1 L increase	1.004 (0.844,1.195)	0.963	0.999 (0.740,1.347)	0.993
TBW, 1 L increase	1.006 (0.946,1.069)	0.859	1.043 (0.933,1.167)	0.458
ECW/ICW, 1 % increase	0.946 (0.820,1.092)	0.452	0.502 (0.333,0.757)	<0.001
ICW/TBW, 1 % increase	1.158 (0.801,1.673)	0.436	6.258 (2.092,18.718)	0.001
ECW/TBW in total, 1 % increase	0.882 (0.607,1.283)	0.512	0.188 (0.068,0.514)	0.001
PhA, 1 degree increase	1.281 (0.817,2.009)	0.281	4.881 (1.854,12.847)	0.001

Results obtained after adjusting for age, gender, comorbidity, and BMI. Abbreviations: aOR, adjusted odds ratio; 95% CI, 95% confidence interval; WBC, white blood cell; AST, aspartate aminotransferase; ALT, alanine aminotransferase; CRP, C–reactive protein; BIA, bioelectrical impedance analysis; SLM, soft lean mass; FFM, fat free mass; SMM, skeletal muscle mass; PBF, percent body fat; BCM, body cell mass; VFA, visceral fat area; SMI, skeletal muscle mass index; ICW, intracellular water; ECW, extracellular water; TBW, total body water; ECW/ICW, extracellular water–to–intracellular water ratio; ICW/TBW, intracellular water–to–total body water ratio; ECW/TBW, extracellular water–to–total body water ratio; PhA, phase angle

Table S3: Interactions of biomarkers and ECW/TBW, PhA on dengue in the study population (n=186)

Parameters	Dengue			
	Interaction with ECW/TBW		Interaction with Phase Angle	
	OR (95% CI)	p-value	OR (95% CI)	p-value
WBC	0.938 (0.800,1.100)	0.433	1.065 (0.896,1.267)	0.473
Neutrophils	1.010 (0.991,1.030)	0.313	0.993 (0.969,1.017)	0.563
Lymphocytes	0.989 (0.964,1.015)	0.412	0.984 (0.965,1.004)	0.115
Hematocrit	1.126 (0.939,1.220)	0.154	0.870 (0.704,1.014)	0.053
Hemoglobin	1.035 (0.989,1.059)	0.094	0.960 (0.935,1.012)	0.058
Platelets	1.006 (0.999,1.013)	0.098	0.991 (0.983,1.001)	0.052
AST	0.998 (0.992,1.003)	0.432	1.004 (0.997,1.010)	0.299
ALT	1.002 (0.996,1.007)	0.523	0.999 (0.993,1.004)	0.612
Urea	0.995 (0.832,1.191)	0.957	1.001 (0.828,1.212)	0.988
Creatinine	1.013 (0.999,1.027)	0.056	0.989 (0.976,1.002)	0.989
CRP	0.993 (0.972,1.014)	0.507	1.001 (0.978,1.022)	0.977
Potassium	0.855 (0.408,1.791)	0.678	1.183 (0.497,2.817)	0.703
Sodium	1.059 (0.963,1.166)	0.236	0.949 (0.850,1.061)	0.358
Chloride	1.027 (0.941,1.122)	0.547	0.991 (0.900,1.092)	0.859

Abbreviations: OR, odds ratio; 95% CI, 95% confidence interval; ECW/TBW, extracellular water–to–total body water ratio; WBC, white blood cell; AST, aspartate aminotransferase; ALT, alanine aminotransferase; CRP, C–reactive protein.

Table S4: Factors associated with length of hospital stay in dengue patients from bivariate regression analysis (n=104)

**Parameters**	**Length of hospital stay**	
	**Dengue subjects**	
	**B (95 %CI)**	**p-value**
Age		
< 60 years	Ref	
≥ 60 years	–0.201 (–1.228,0.826)	0.699
Gender		
Male	Ref	
Female	–0.360 (–1.185,0.464)	0.388
Comorbidity		
No	Ref	
One or more	1.471 (0.535,2.406)	0.002
BMI		
Non-obese (< 25)	Ref	
Obese (≥ 25)	0.750 (–0.337,1.837)	0.174
Dengue severity		
Dengue without warning signs	Ref	
Dengue with warning signs	–0.626 (–1.469,0.218)	0.144
**Laboratory parameters**		
WBC, $1\times10^3/\mu L$ increase	0.247 (0.103,0.392)	<0.001
Neutrophils, 1 % increase	0.051 (0.029,0.072)	<0.001
Lymphocytes, 1 % increase	–0.069 (–0.096,–0.042)	<0.001
Hematocrit, 1 % increase	0.010 (–0.070,0.091)	0.799
Hemoglobin, 1 g/L increase	–0.004 (–0.028,0.019)	0.724
Platelets, $1\times10^3/\mu L$ increase	0.014 (0.007,0.022)	0.010
AST, 1 IU/L increase	0.0004 (–0.003,0.002)	0.741
ALT, 1 IU/L increase	0.001 (–0.002,0.004)	0.510
Urea, 1 mmol/L increase	0.426 (0.168,0.683)	0.002
Creatinine, 1 µmol/L increase	0.019 (0.001,0.036)	0.039
CRP, 1 mmol/L increase	0.033 (–0.009,0.076)	0.123
Potassium,1 mmol/L increase	–0.715 (–1.626,0.195)	0.122
Sodium, 1 mmol/L increase	0.074 (–0.017,0.164)	0.109
Chloride, 1 mmol/L increase	0.078 (–0.048,0.205)	0.222
**BIA parameters**		
Protein, 1 kg increase	0.048 (–0.177,0.273)	0.672
Mineral, 1 kg increase	0.171 (–0.470,0.811)	0.599
SLM, 1 kg increase	0.011 (–0.037,0.060)	0.641
FFM, 1 kg increase	0.011 (–0.035,0.057)	0.640
SMM, 1 kg increase	0.016 (–0.059,0.090)	0.679
PBF, 1 percent increase	0.012 (–0.035,0.059)	0.609
BCM, 1 kg increase	0.017 (–0.053,0.082)	0.677
VFA, 1 cm^2 increase	0.007 (–0.006,0.021)	0.270
SMI, 1 kg/m^2 increase	0.160 (–0.231,0.550)	0.419
ICW, 1 L increase	0.021 (–0.076,0.117)	0.675
ECW, 1 L increase	0.054 (–0.126,0.234)	0.555
TBW, 1 L increase	0.015 (–0.048,0.079)	0.483
ECW/ICW, 1 % increase	0.037 (–0.117,0.191)	0.635
ICW/TBW, 1 % increase	–0.091 (–0.486,0.305)	0.650
ECW/TBW in total, 1 % increase	0.095 (–0.306,0.496)	0.639
PhA, 1 degree increase	0.029 (–0.452,0.510)	0.905
ΔICW, 1 L increase	0.422 (–0.799,1.643)	0.491
ΔECW, 1 L increase	0.338 (–1.129,1.805)	0.646

ΔTBW, 1 L increase	0.225 (–0.514,0.965)	0.544
ΔECW/TBW total, 1 % increase	0.355 (–0.731,1.440)	0.515

Abbreviations: B, Regression coefficients; 95% CI, 95% confidence interval; Ref, reference; WBC, white blood cell; AST, aspartate aminotransferase; ALT, alanine aminotransferase; CRP, C–reactive protein; BIA, bioelectrical impedance analysis; SLM, soft lean mass; FFM, fat free mass; SMM, skeletal muscle mass; PBF, percent body fat; BCM, body cell mass; VFA, visceral fat area; SMI, skeletal muscle mass index; ICW, intracellular water; ECW, extracellular water; TBW, total body water; ECW/ICW, extracellular water–to–intracellular water ratio; ICW/TBW, intracellular water–to–total body water ratio; ECW/TBW, extracellular water–to–total body water ratio; PhA, phase angle, ΔICW, the difference of ICW values between the day of admission and defervescence; ΔECW, the difference of ECW values between the day of admission and defervescence; ΔTBW, the difference of TBW values between the day of admission and defervescence; ΔECW/TBW total, the difference of ECW/TBW total values between the day of admission and defervescence.

Table S5: Interactions of biomarkers and ECW/TBW, PhA on LOS in dengue patients (n=104)

**Parameters**	**Length of hospital stay**			
	**Interaction with ECW/TBW**		**Interaction with Phase Angle**	
	**B (95% CI)**	**p-value**	**B (95% CI)**	**p-value**
WBC	0.002 (–0.186,0.191)	0.980	–0.064 (–0.281,0.152)	0.555
Neutrophils	0.150 (–0.021,0.025)	0.881	–0.001 (–0.025,0.024)	0.976
Lymphocytes	0.012 (–0.019,0.044)	0.433	–0.025 (–0.056,0.006)	0.114
Hematocrit	–0.068 (–0.164,0.028)	0.163	–0.068 (–0.165, 0.029)	0.170
Hemoglobin	0.019 (–0.004,0.043)	0.102	–0.024 (–0.053,0.006)	0.117
Platelets	–0.005 (–0.013,0.002)	0.167	0.006 (–0.002,0.014)	0.140
AST	0.0001 (–0.003,0.003)	0.945	–0.0002 (–0.003,0.003)	0.903
ALT	–0.0001 (–0.004,0.003)	0.964	0.0001 (–0.004,0.005)	0.960
Urea	–0.038 (–0.298,0.221)	0.769	0.034 (–0.273,0.341)	0.825
Creatinine	0.002 (–0.016,0.021)	0.803	–0.002 (–0.025,0.022)	0.884
CRP	0.056 (–0.016,0.026)	0.057	–0.028 (–0.076,0.020)	0.253
Potassium	0.132 (–0.667,0.930)	0.744	–0.444 (–1.596,0.708)	0.445
Sodium	–0.140 (–0.280, 0.003)	0.052	0.102 (–0.069,0.273)	0.240
Chloride	–0.070 (–0.204,0.064)	0.299	0.077 (–0.080,0.234)	0.331

Abbreviations: B, Regression coefficients; 95% CI, 95% confidence interval; ECW/TBW, extracellular water–to–total body water ratio; WBC, white blood cell; AST, aspartate aminotransferase; ALT, alanine aminotransferase; CRP, C–reactive protein.
